# Metformin therapy in pediatric type 2 diabetes mellitus and its comorbidities: A review

**DOI:** 10.3389/fendo.2022.1072879

**Published:** 2023-02-06

**Authors:** Haifa Alfaraidi, M. Constantine Samaan

**Affiliations:** ^1^ College of Medicine, King Saud bin Abdulaziz University for Health Sciences, Ministry of National Guard Health Affairs, Riyadh, Saudi Arabia; ^2^ Department of Pediatrics, King Abdullah Specialized Children’s Hospital, King Abdulaziz Medical City, Ministry of National Guard Health Affairs, Riyadh, Saudi Arabia; ^3^ King Abdullah International Medical Research Center, Ministry of National Guard Health Affairs, Riyadh, Saudi Arabia; ^4^ Department of Pediatrics, McMaster University, Hamilton, ON, Canada; ^5^ Division of Pediatric Endocrinology, McMaster Children’s Hospital, Hamilton, ON, Canada; ^6^ Department of Health Research Methods, Evidence and Impact, McMaster University, Hamilton, ON, Canada; ^7^ Michael G. De Groote School of Medicine, McMaster University, Hamilton, ON, Canada

**Keywords:** metformin, type 2 diabetes, youth, management, pediatric type 2 diabetes

## Abstract

Type 2 diabetes (T2D) rates in children and adolescents are rising globally. T2D is a complex and aggressive disease in children with several comorbidities, high treatment failure rates, and insulin needs within a few years from diagnosis. While myriads of pharmacotherapies are licensed to treat adults with T2D, treatments accessible to children and adolescents have been limited until recently. Metformin is an old drug with multiple beneficial metabolic health effects beyond glycemic control. This review discusses Metformin’s origins, its mechanisms of action, and evidence for its use in the pediatric population to treat and prevent T2D. We also explore the evidence for its use as an obesity therapy, which is the primary driver of T2D, and T2D-driven comorbidities. While emerging therapies create new horizons for managing pediatric T2D, Metformin remains an inexpensive and safe part of the treatment plans of many T2D children globally for its beneficial metabolic effects.

## Introduction

Pediatric type 2 diabetes (T2D) has become a global public health concern driven by childhood obesity (1–4). Multiple biosocial drivers contribute to the intertwining of both diseases (1, 5). Over the past three decades, T2D has increased steadily in children (6), with a 7.1% annual increase in incidence rates (3) and a 95% increase in prevalence in the USA over almost two decades (7). Recent global data suggest incidence rates of up to 31-94 per 100,000 per year and prevalence of up to 160-5,300 per 100,000 in high-risk populations, such as Indigenous and African youth (8). While initially described as a disease of Indigenous children, current data reveal a surge in T2D across different ethnic groups (9, 10). There are currently no population-based screening programs for T2D in children; the disease remains uncommon in children, with most cases of new-onset diabetes being type 1 diabetes. This pattern is coupled with the 30% reproducibility of one of the primary screening tests in children–the oral glucose tolerance test (11). The use of glycated hemoglobin A1c (HbA1c) has been purposed for monitoring glycemic control in these children rather than establishing the diagnosis alone (12, 13). The more reproducible test, fasting glucose, is not very practical in children for obvious reasons and does miss those who become hyperglycemic after a carbohydrate challenge.

As up to 50% of children with T2D are asymptomatic at presentation and are mostly diagnosed when screened because they have obesity, the current prevalence and incidence rates for T2D likely underestimate the true scale of the disease in children (9, 14).

T2D presents as an aggressive disease with multiple comorbidities, and patients have a progressive failure of β-cell function and progress to insulin dependence within 3-5 years post-diagnosis. This rapid deterioration of β-cell function is more rapid in children when compared to adults with T2D (15–19).

Several conditions are associated with T2D including obesity, dyslipidemia, hypertension, obstructive sleep apnea, polycystic ovary syndrome, and non-alcoholic fatty liver disease (20–24). Microvascular complications manifest as nephropathy, neuropathy, and retinopathy and present much earlier than those in children with type 1 diabetes (25).

T2D impacts the children’s longevity and quality of life, and the premature mortality seen in this population is a significant concern (26).

While initial approaches to treatment involved using lifestyle to manage diabetes, it has become clear that this approach needs supplementation with pharmacotherapy to establish and maintain glycemic control (27). So far, pharmacotherapies used to treat T2D include metformin, insulin, sodium-glucose cotransporter-2 (SGLT-2) inhibitors, and glucagon-like peptide-1 (GLP-1) receptor agonists (28–31). This review focuses on the use of Metformin in the management of T2D patients.

## Metformin: an ancient drug

Metformin is the most widely prescribed pharmacotherapeutic agent to treat T2D globally (32–35). Metformin is a biguanide derived from a guanidine, galegine, from the French lilac or goat’s rue (*Galega officinalis)*, which was used for centuries to treat diabetes (36). Metformin hydrochloride (HCl) is hydrophilic and has a molecular weight of 165.63 kDa (37). The molecular formula of Metformin HCl, the compound used in clinical preparations, is C_4_H_11_N_5_ • HCl (37). Metformin is hydrophilic and has a molecular weight of 129.16 kDa (38). The molecular formula of Metformin is C_4_H_11_N_5_ (38). For the remainder of the paper, the use of Metformin assumes the use of Metformin HCl.

Advances in chemistry and new drug manufacturing methods pioneered almost a century ago led to the synthesis of Metformin in 1922 as dimethylbiguanide (39). However, it did not come into broad use to treat diabetes initially. One of the most critical biomedical discoveries of the 20^th^ century–insulin in 1921, overshadowed the use of other medications to treat diabetes (40). In 1957, Metformin was studied by Dr. Jean Sterne, who investigated its antihyperglycemic properties and called it Glucophage (glucose eater), a name that still resonates today as a brand name for metformin (41). Although the higher potency of other biguanides, such as phenformin, led to their prioritization in clinical trials (42), the development of lactic acidosis led to early discontinuation of the trials and the interest in Metformin was reignited (43, 44). While less potent than other guanidines, Metformin did possess a better safety profile (45). Furthermore, the U.K. Prospective Diabetes Study further supported the use of Metformin in T2D, with improved glycemic outcomes and decreased morbidity and mortality (46). These discoveries elevated Metformin to become the first-line therapy for adults with T2D (46, 47).

Metformin HCl was licensed in the United Kingdom in 1958 and in 1972 in Canada (48). The U.S. Food and Drug Administration (FDA) only approved the use of Metformin for T2D treatment in adults in December of 1994 and in children ≥10 years old with T2D in December of 2000 (49). Extended-release metformin formulations were subsequently approved (50), and the first extended-release liquid formulation followed more recently (51, 52). The availability of liquid formulations offers an alternative to Metformin tablets that may be large or hard to swallow for pediatric patients, leading to decreased adherence. Additionally, when evaluating the taste of liquid Metformin formulations, they were preferred compared to crushed Metformin tablets (53). Therefore, liquid Metformin may improve adherence to treatment in the pediatric population. However, the liquid form may not be readily available in all parts of the world.

## Pharmacokinetics and pharmacodynamics

Metformin is taken orally. The typical doses to treat diabetes for immediate-release preparations are 500-2,000 mg/day. The leading site of metformin absorption is the small intestine in the duodenum and jejunum *via* the action of plasma membrane monoamine transporter (PMAT) in intestinal cells, with a minor contribution of the colon (54, 55).

Taken orally, Metformin has a bioavailability of up to 60%, and it is absorbed within 6 hours (56). After absorption, Metformin does not bind to plasma proteins and is distributed to the liver (57–59). The non-absorbed Metformin accumulates at the distal small intestine, and it is then eliminated in the feces (60).

Metformin has a mean elimination plasma half-life of around 5 hours. Its onset of action is at around 1.5 hours, and the total duration of action is 16-20 hours (61). The therapeutic concentration of Metformin has been debated, and there are limitations in the literature about the levels and their methods of measurement at clinically relevant doses. However, a widely accepted circulating level at therapeutic doses (20 mg/kg/day) is 10-40 μmol/L (62, 63). Metformin is excreted unchanged *via* renal tubular excretion as the major route for elimination (64).

The extended-release formulation is based on the combination of Metformin with polymer delivery systems that allow the tablet to absorb water and expand to 150% of its original size within 15 minutes of ingestion, anchoring it in the stomach (65, 66). The pill slowly releases Metformin into the small intestine over 8-9 hours. When the Metformin is released, the tablet’s excipients disintegrate and are excreted in the feces at around 15 hours post-ingestion. The main advantage of extended-release Metformin is the potential for improved adherence to treatment in children and reduced gastrointestinal side effects. This metformin formulation is administered once daily, and the typical dose of these preparations is 1000-2000 mg/day, with a half-life of 6.5 hours and actions on plasma glucose for 24 hours (65, 67) (68).

## Mechanisms of action

**Figure 1:**
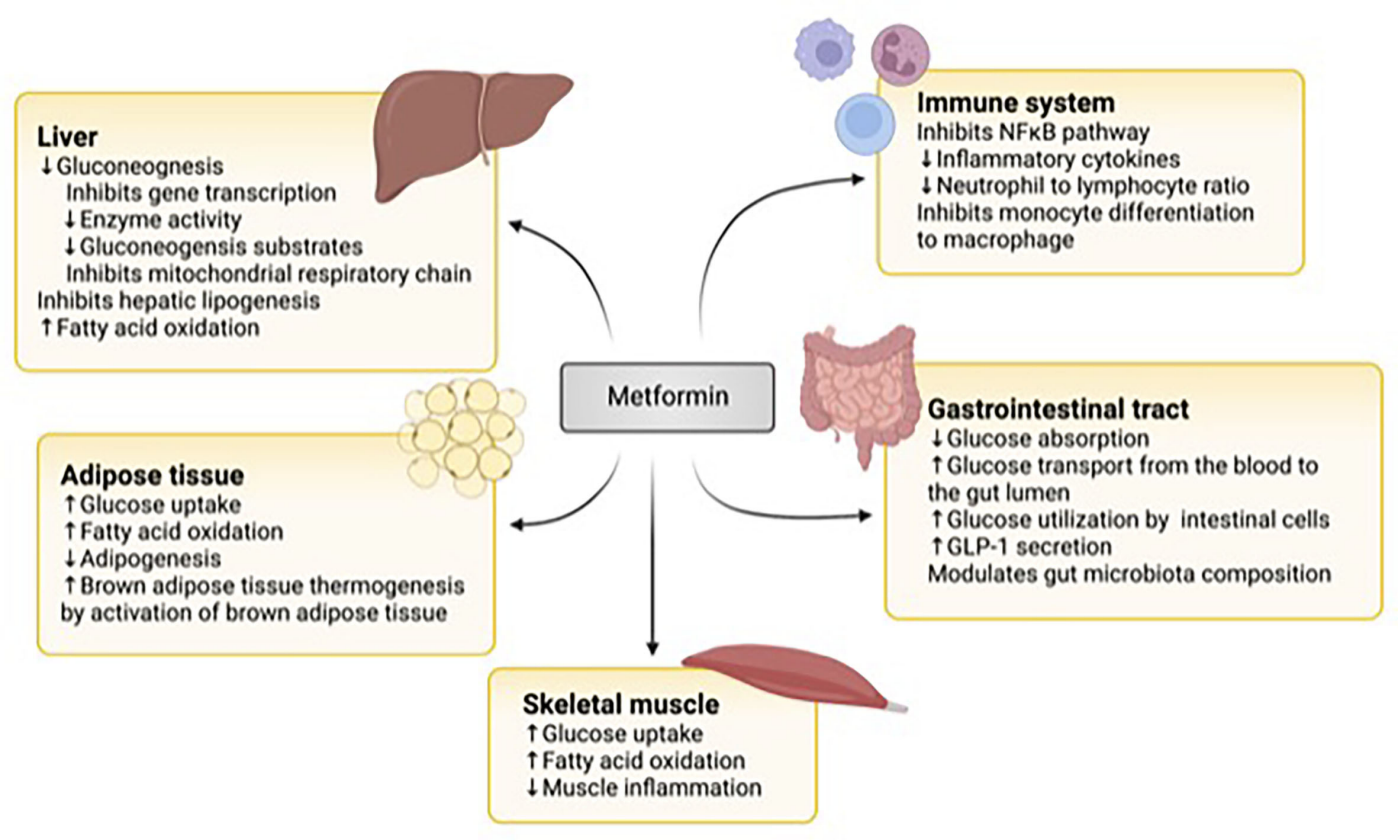
The mechanisms of action of metformin. In the liver, metformin downregulates gluconeogenesis by inhibiting gene transcription, gluconeogenic enzyme activity and substrates, and the mitochondrial respiratory chain. It also inhibits lipogenesis and upregulates fatty acid oxidation. In the adipose tissue, it increases glucose uptake and fatty acid oxidation and brown adipose tissue while downregulating adipogenesis. In skeletal muscle, metformin stimulates glucose uptake and fatty acid oxidation. Metformin inhibits the nuclear factor KB (NFKB) pathway in immune cells and the differentiation of monocytes to macrophages. It also lowers inflammatory cytokine production and the neutrophil to lymphocyte chain. In the gastrointestinal tract, it modulates the gut microbiome, decreases glucose absorption from the intestin, It also increases glucose transport from the blood to the gut lumen, glucose utilization by intestinal cells, and glucagon peptide-1 (GLP-1) secretion. Figure created with BioRender.com.

Metformin exerts its glucose-lowering effect through various mechanisms in different organs (**Figure 1**).

The liver is one of the important target organs of metformin actions. Metformin reduces hepatic glucose output by inhibiting gluconeogenesis (69, 70). While Metformin inhibits complex 1 of the respiratory chain in the mitochondria leading to an increase in adenosine monophosphate (AMP) and activation of AMP-activated protein kinase (AMPK), which in turn inhibits hepatic gluconeogenesis, the concentration of Metformin needed to achieve such effects in humans is supra-therapeutic (71, 72).

Additionally, Metformin inhibits hepatic gluconeogenesis by AMPK-independent mechanisms (73). These mechanisms include inhibiting gene transcription (73) and activity of enzymes in the gluconeogenic pathway (74), in addition to decreasing substrates available for gluconeogenesis such as lactate and glycerol (75, 76). Metformin also inhibits hepatic lipogenesis and increases fatty acid oxidation (77, 78).

Metformin’s additional actions include increasing glucose uptake and fatty acid oxidation in skeletal muscle and adipose tissue (78–82). It also reduces adipogenesis and activates brown adipose tissue leading to enhanced thermogenesis (83).

More recently, the effect of Metformin on the gut has been recognized (84). Metformin decreases the absorption of glucose from the intestine. It increases glucose transport from the blood into the lumen of the intestine (85–87), in addition to increasing glucose utilization by intestinal cells (88). Metformin also promotes the secretion of Glucagon-Like Peptide-1 (GLP-1) from the upper intestine, augmenting glucose-dependent insulin secretion (89, 90). Furthermore, Metformin influences the gut microbiome by promoting the growth of beneficial species that degrade mucin and generate short-chain fatty acids (91, 92). Animal studies have shown that mice given a metformin-modulated gut microbiome have improved glucose tolerance (92).

Metformin downregulates inflammation by inhibiting the NFϰB pathway and decreasing the secretion of inflammatory cytokines (93, 94). It also affects the proportion of white blood cells by reducing the ratio of neutrophils to lymphocytes (93) and inhibits the differentiation of monocytes to macrophages (95).

## Treating type 2 diabetes with metformin 

Lifestyle interventions encompassing healthy eating and physical activity recommendations need to be provided to children with T2D in a culturally and developmentally appropriate manner (96). While the exact endpoints of lifestyle interventions may not be easily achievable and are not solely focused on glycemic control, a suggested target is at least a 7% weight loss, presumed to improve metabolic markers such as HbA1c and C-peptide levels (97), and may help reduce the risk of microvascular complications (98). However, the aggressive nature of T2D and the high treatment failure rates have put a particular urgency on efforts to identify adjunct therapies to lifestyle interventions that may improve outcomes. 

Currently, Metformin is the first pharmacotherapeutic choice for children with T2D with metabolic stability, including being asymptomatic and with an HbA1c <8.5% and blood glucose levels <250 mg/dL (<13.9 mmol/L) (96) (**Tables 1**, **2**). In the presence of symptomatic hyperglycemia with polyuria, polydipsia, and weight loss, with biochemical deterioration and HbA1c >8.5% and blood glucose >250 mg/dL (≥14.0 mmol/L) and no acidosis, both Metformin and basal insulin are initiated (96). In general, Metformin treatment reduces HbA1c by 1-2% (99, 102).

**Table 1:** Metformin Use in Children with type 2 diabetes.

Author, year	Study Design	Population	Intervention	Study Duration	Outcomes	Results
Jones et al, 2002 (99)	Randomized controlled-trial, double blinded	-Youth aged 8-16 years with T2D -FPG: 7-13.3 mmol/L -HbA1c > 7.0% -Stimulated C-peptide > 0.5 mmol/L - BMI >5oth% for age n=82	Metformin 1,000 mg BID vs placebo	eeks	Glycemic control: - Fasting plasma glucose - HbA1c	-Fasting plasma glucose decreased by a mean change of 2.4 (+/- 0.5) mmol/L in the Metformin group compared with a 1.2 (+/- 0.5) mmol/L increase in the placebo group. -Baseline HbA1c improved from 8.2% (+/- 1.3) to 7.5% (+/-0.2) in the Metformin group. -Baseline HbA1c improved from 8.9% (+/- 1.4) to 8.6% (+/- 0.2) in the placebo group.
Gottschalk et al, 2007 (100)	Randomized single-blind comparative study	-Children and adolescents aged 8-17 years with T2D -HbA1c 7.2% to 11.9% n= 263	-Metformin 500-1,000 mg BID -Glimepiride 1-8 mg once daily	26 weeks	Change in HbA1c from baseline to 12 weeks and 24 weeks	Mean change in HbA1c - At 12 weeks: – 0.69% with glimepiride vs – 0.76% with Metformin (p=0.75) -At 24 weeks: –0.70% with Glimepiride vs – 0.85% with Metformin (p= 0.54) Similar events of hypoglycemia inn both groups Significant weight increase in the Glimepiride group but not in the Metformin group
TODAY Study, 2012 (27)	Randomized controlled trial, 3 arms	- Youth aged 10-17 years - T2D less than 2 years - BMI > 85th % for age and sex - Fasting c-peptide > 6.0 ng/mL - Negative diabetes autoantibodies n=699	- Metformin plus rosiglitazone - Metformin with lifestyle interventions - Metformin alone	Minimum 2 years Mean 3.8 years	Time to treatment failure: - Elevated HbA1c > 8% for 6 months - Metabolic decompensation as inability to wean of insulin	Failure rates: Metformin plus rosiglitazone: 38.6% Metformin plus lifestyle interventions: 46.6% Metformin alone: 51.7%
Marcus et al, 2017 (97)	Sub analysis of the TODAY study group	- Youth from the initial TODAY study group n= 595	- Metformin plus rosiglitazone - Metformin with lifestyle interventions - Metformin alone	24 months	Change in % overweight: BMI minus BMI at 50th% for sex and age, divided by BMI at 50th% then multiplied by 100 Change in BMI	-Metformin alone and Metformin plus lifestyle had a favorable effect on % overweight at 12 and 24 months compared to Metformin and Rosiglitazone. - Around 30% of participants in the Metformin alone and the Metformin and lifestyle interventions groups achieved 7% or more weight loss. -There was no significant difference between Metformin and Metformin plus lifestyle interventions for % overweight and for BMI at 12 and 24 months.
Matsuura et al, 2019 (101)	Open-label, non-randomized trial	-Children and adolescents from 6-17 years of age with T2D - HbA1c 7.1%-12.0% n=37	-Metformin-naïve patients (treated by diet-exercise, sulfonylurea, or a-glucosidase inhibitor) before switching to Metformin -Already-on Metformin group (<750 mg/day for 12 weeks) -Both groups were started/continued on Metformin, dose titrated upwards to maximum of 2,000 mg/day based on glycemic control	52 weeks: 24 weeks of treatment and 28-week extension period	-Change in HbA1c from baseline to 24-weeks and to 52-weeks	Mean change in A1c: -At 24 weeks: – 0.81% (+/- 1.29); 95% CI (– 1.24 to – 0.37) -At 52 weeks: – 0.46% (+/- 1.13); 95% CI (– 0.83 to – 0.08)

**Table 2:** Summary of Metformin Indications.

**Metformin in type 2 diabetes** •Metformin is considered first line treatment as monotherapy or in combination with insulin. •Metformin provides sustained glycemic control in around 50% of pediatric individuals with T2D. •No current evidence to suggest that Metformin preserves β-cell function with new onset T2D.
**Metformin for treatment of obesity** •Metformin improves anthropometric indices such as BMI, weight, and waist circumference. •These effects are not sustained and typically fade after 1-2 years. •Metformin may promote weight loss in a subset of obese youth with T2D.
**Metformin for treatment of PCOS** •Metformin has a beneficial effect on weight, BMI, and insulin resistance in adolescent girls with PCOS. •Metformin reduces free testosterone levels and improve hirsutism and menstrual irregularity in adolescent girls with PCOS. •The use of Metformin in patients with co-existing T2D and PCOS provides the added benefit of ameliorating clinical and biochemical features of PCOS and improving glycemic control.
**Metformin for treatment of NAFLD** •Metformin does not affect liver enzymes in youth with NAFLD. •Metformin may improve histologic and radiographic findings associated with NAFLD. •As NAFLD may co-exist with T2D, the stability of liver enzymes with Metformin is reassuring.
**Metformin for the prevention of T2D** •Most studies assessing the role of Metformin in preventing T2D in youth have focused on indices of insulin resistance rather than progression to overt T2D. •Evidence regarding the effect of Metformin on insulin sensitivity and glycemic control in obese youth with markers of insulin resistance is inconsistent. •Long-term studies focusing on prevention of progression to T2D are required.

**Abbreviations:** T2D, type 2 diabetes; BMI, body mass index; PCOS, polycystic ovary syndrome; NAFLD, non-alcoholic fatty liver disease.

A minority of patients with T2D present in diabetic ketoacidosis (DKA) at diagnosis (103). In these patients, conventional DKA management with insulin is initiated to reverse the acidosis; then Metformin is started in addition to subcutaneous insulin therapy for a few weeks and then a decision is made as to whether insulin can be weaned or stopped with continued Metformin treatment (96).

Because of its gastrointestinal side effects, Metformin should be started at a low dose with gradual titration, depending on tolerance. The typical metformin regimen involves a starting dose of 250-500 mg/day, with the weekly escalation of the dose up to a maximum of 1,000 mg twice daily or 850 mg three times per day (96, 104, 105). Extended-release formulations can be prescribed to reduce gastrointestinal side effects, or Metformin can be taken with meals.

Even though Metformin has been approved for use in pediatric T2D for decades, prospective studies in this population are limited, and trials of new medications have used them as add-ons to Metformin with or without insulin.

A randomized controlled trial of 16 weeks compared Metformin to a placebo in youth aged 10-16 with T2D. Metformin lowered HbA1c and fasting plasma glucose when compared to placebo (99).

Another study from Japan included youth with T2D and found that Metformin decreased HbA1c by 0.81% at 24 weeks and 0.46% at 52 weeks (101). While gastrointestinal side effects were widespread in almost all patients, no serious adverse events necessitated Metformin’s discontinuation over the 52-week study period (101).

Compared to glimepiride, a sulfonylurea, Metformin achieved similar reductions in HbA1c in those aged 8-17 years with T2D. While the incidence of hypoglycemia was similar in both groups, Metformin led to less weight gain compared to glimepiride (100).

Significant insights into the role of Metformin in pediatric T2D management were gleaned from the seminal TODAY study, a randomized controlled clinical trial that included 699 children with T2D. The trial compared Metformin with or without lifestyle interventions and Metformin plus rosiglitazone. The primary outcome was the need to use insulin due to failure in achieving glycemic control, defined as an HbA1c <8% for at least six months (106).

At a median of two months of metformin therapy during the run-in period, Metformin led to 91% of participants achieving an HbA1c <8%, with 78% achieving an HbA1c level less than 7% and 46% having an HbA1c level less than 6% (107). The benefits of Metformin waned over time, with only 49.3% for Metformin alone and 53.4% for the Metformin plus lifestyle intervention achieving durable glycemic control after four years (27).

Importantly, an HbA1c of <6.3% at randomization was associated with durable glycemic control (108). In participants with deteriorating glycemic control, a decline in β-cell function rather than reduced insulin sensitivity was noted (18, 109). Data from the Pediatric Diabetes Consortium T2D Registry confirmed this association of lower HbA1c at diagnosis with maintained glycemic control ≥2 years post-diagnosis (110).

Importantly, in youth with recent-onset T2D or impaired glucose tolerance, neither insulin glargine followed by metformin nor metformin alone preserved β-cell function (111). However, discontinuation of therapy resulted in worsening glycemic control, reflected by HbA1c, fasting and 2-hour post prandial glucose, as well as a decline in β-cell function after a 9-month washout period (112).

Future research efforts must concentrate on understanding Metformin’s glycemic effects and potential benefits in preventing or decreasing diabetes-related complications and cardiovascular events.

## Metformin therapy of type 2 diabetes-related comorbidities 

### Obesity

Obesity is a significant risk factor for T2D in children, and the prevalence of overweight and obesity is close to 75% in youth with T2D (113). Metformin has been shown to reduce weight, BMI, and waist circumference (114–126). The weight loss associated with Metformin is usually modest and results from its action on the gastrointestinal tract leading to decreased appetite. Additionally, Metformin has a positive effect on markers of insulin resistance, such as fasting plasma glucose, fasting insulin levels, and homeostasis model assessment-estimated insulin resistance (HOMA-IR) (114, 116–118, 121, 122, 125). Interestingly, Metformin may improve weight, BMI, waist circumference, total adiposity, and abdominal adiposity in prepubertal children born small for gestational age (127). 

Metformin reduces BMI by around 1.4 kg/m^2^ (125, 126, 128–131), and most clinical trials were relatively short, following participants for 6-12 months (114–116, 119–122). Most weight loss occurs in the first six months of treatment, tends to slow down, and is not necessarily sustained after treatment cessation (128–132). In an 18-month multicenter randomized controlled trial that included 42 participants (66% females) with a median age of 13, BMI improved in the metformin group at 6 and 9 months. Yet, it returned to baseline at the end of the 18- month study period (133). Additionally, an open-label 18- month extension of the study confirmed the lack of a sustainable effect of Metformin on BMI in adolescents with longer-term follow up (134). Interestingly, while the effect of Metformin on weight and BMI may not be sustained, youth with obesity treated with Metformin had less weight and BMI gain when compared to those who did not receive Metformin (135, 136).

While most studies demonstrate improvement in weight or BMI on treatment (114–122), trials of longer duration that may need to be combined with co-interventions are needed to address its long-term effects on body mass and metabolic health.

In the TODAY study, BMI at two years of treatment increased by 1.6 and 1.4 kg/m^2^ in the metformin and metformin plus lifestyle intervention groups, respectively (97). However, a subgroup of patients, representing about one-third in each of the metformin and the metformin plus lifestyle intervention groups, achieved a weight loss of ≥7% at 12 months and 24 months. This weight loss was associated with improved cardiometabolic health markers such as HbA1c, systolic blood pressure and lipid profile (97). These results suggest that a subgroup of youth with T2D may benefit from treatment with weight reduction and improved glycemic control and cardiometabolic markers. Defining this subgroup in detail is critical to personalize care and improve outcomes in pediatric T2D (**Table 2**).

### Polycystic ovary syndrome

Polycystic ovary syndrome (PCOS) is characterized by menstrual irregularities with anovulation or oligo-ovulation with clinical and biochemical hyperandrogenism (137–141). The prevalence of PCOS in girls with T2D is close to 20% (21), compared with up to 11% in the general pediatric population (142). Hyperinsulinism and insulin resistance are essential drivers in the pathophysiology of PCOS (143–145), and this may be related to elevated insulin levels promoting luteinizing hormone secretion from the pituitary gland, with the combined effect of insulin and luteinizing hormone stimulation of the ovarian theca cells to produce androgens (146–148). Although obesity is a significant risk factor for PCOS, a proportion of adolescent girls with PCOS have a normal BMI and evidence of insulin resistance (145, 149, 150).

Metformin has been considered as a treatment option for PCOS. Small randomized controlled studies demonstrated that Metformin promoted a decrease in weight and BMI (151–153) and improved insulin sensitivity (152, 153). Metformin also had favorable effects on clinical and biochemical hyperandrogenism, with decreased free testosterone levels (151, 152, 154, 155) and improved hirsutism (153, 155) and menstrual irregularity (152, 154). Compared to combined oral contraceptive pills (OCPs), one of the main strategies employed for treating PCOS, Metformin is superior to OCPs in reducing BMI and improving insulin sensitivity (156). On the other hand, OCPs resulted in a more significant improvement in menstrual irregularity (156). Metformin and OCPs had an equivalent positive effect on reducing testosterone levels and improving hirsutism (156).

Given the high prevalence of PCOS in girls with T2D, using Metformin in this population may have the added benefit of improving the clinical and biochemical features of PCOS in addition to reducing weight, improving insulin sensitivity, and achieving glycemic targets (**Table 2**).

### Non-alcoholic fatty liver disease

Non-alcoholic fatty liver disease is a spectrum of hepatic disorders that range from steatosis to steatohepatitis and can ultimately progress to cirrhosis. The steatosis is caused by abnormal hepatic lipid metabolism, with insulin resistance and hyperinsulinemia playing a central role (157–160). Fat mass expansion and insulin resistance in obesity lead to the mobilization of free fatty acids from the adipose tissue, which eventually enter the liver, exceeding its oxidation capacity (157–161). Additionally, hyperinsulinemia promotes hepatic lipogenesis (157–160). This combination of increased fatty acid influx, decreased fatty acid oxidation, and increased hepatic triglyceride synthesis leads to hepatic steatosis (157–160). About 50% of youth with T2D have NAFLD, and the degree of dysglycemia correlates with the severity of NAFLD (162, 163).

The current mainstay of therapy for NAFLD is lifestyle intervention, which has been shown to improve both radiologic and biochemical evidence of steatosis (164). Adding Metformin to lifestyle change, compared to placebo or vitamin E supplementation, did not significantly improve liver enzymes (165–167). However, while Metformin does not impact liver enzyme levels, including alanine transaminase (ALT) and aspartate transaminase (AST), it may help lessen histologic findings such as lobular inflammation and hepatocellular ballooning (165, 167), as well as reduce fatty deposits in the liver on ultrasound (166, 168) (**Table 2**). Although current evidence does not support the use of Metformin as a treatment for NAFLD, the stable liver enzymes profile with its use is reassuring (165–167).

## Off-label use of metformin in children younger than 10 years of age and pre-adolescents

There is limited evidence that suggests that Metformin is safe and effective in improving weight or BMI as well as other metabolic markers of obesity in children younger than 10 years of age.

A randomized trial evaluating the use of Metformin to treat obesity in prepubertal children recruited 80 participants, who were randomized to either Metformin or placebo (169). At 6-months, the Metformin arm had a lower BMI z-score, as well as an improved quantitative insulin sensitivity check index (QUICKI) and a higher leptin-to-adiponectin ratio compared to the placebo arm, with no serious adverse events or cases of lactic acidosis (169). Similar results were reported by another study that included 18 prepubertal children, with improvement in weight and BMI SDS as well as the fat mass after 24 months of therapy (170). However, due to the small sample size, the study was underpowered to detect a statistically significant difference (170). In obese pre-adolescents with hyperinsulinemia, metformin reduced BMI, BMI SDS, weight, HOMA-IR, fasting insulin, and fasting glucose (122). Yet, there was no significant change in first-phase insulin secretion or insulin sensitivity by clamp studies (122).

Metformin may also have a positive effect on the body composition and metabolic profile of children with a history of being small for gestational age (SGA), given their risk of obesity and insulin resistance (127). A small pilot study that recruited 23 children 6-9 years of age with a history of SGA and catch-up growth with increased visceral fat were randomized to Metformin (n=6) and placebo (n=17) (127). After 24 months, the Metformin arm participants had lower weight and BMI SDS as well as lower total and abdominal fat compared to the control arm (127). Additionally, HOMA-IR improved in the Metformin group (127). There is a need for adequately powered randomized clinical trials to address the role of metformin in managing obesity in this age group.

## Side effects and contraindications

Metformin has a favorable safety profile. The most common metformin-related side effects are gastrointestinal including diarrhea, abdominal pain, bloating, nausea, and loss of appetite. These side effects occurred in around 50% of participants in the TODAY study (27). Gastrointestinal side effects are typically temporary and tend to improve with time (27, 129, 131). These symptoms can be mitigated by gradual dose escalation, ingestion of Metformin alongside meals, or the administration of extended-release preparations.

Metformin has been linked with vitamin B12 deficiency, most likely due to its effect on the gastrointestinal tract (171–173). The most plausible mechanism is the alteration in the calcium-dependent binding of the intrinsic factor and vitamin B12 complex with the receptors in the terminal ileum (174, 175). However, small short-term studies that included pediatric patients on Metformin for various indications did not demonstrate an association between metformin and vitamin B12 deficiency (176, 177).

Serious adverse events related to Metformin, namely hypoglycemia and lactic acidosis, are rare. Metformin’s risk of hypoglycemia is very low, when used as monotherapy and when patients consume carbohydrates regularly (105). Hypoglycemia in metformin therapy typically occurs when insulin or other hypoglycemic medications are also used.

Lactic acidosis is a concerning side effect associated with Metformin in adults and occurs in the presence of hypoperfusion or hypoxemia (178). The risk of lactic acidosis with Metformin is negligible in children and adolescents on Metformin in obesity (129, 131). In the TODAY study, there was only one case of lactic acidosis that was deemed unrelated to metformin use (27). Additionally, there were no clinically significant cases of lactic acidosis reported in two other trials that included youth with T2D on metformin (99, 101). Therefore, Metformin does not seem to cause lactic acidosis in pediatric T2D patients. However, it can certainly occur with a concurrent illness or dehydration that may drive lactic acid build-up.

Contraindications to Metformin include significant cardiac impairment resulting in hypoperfusion and hypoxia (179). Additionally, significant liver impairment is another contraindication (37).

Metformin can be used safely in youth with elevated liver enzymes in the context of NAFLD, provided liver function is preserved (165–167). Until recently, Metformin was also contraindicated for renal impairment. However, the FDA has updated its labelling to indicate that Metformin can be used safely in mild and some cases of moderate renal impairment (180). Metformin is contraindicated when eGFR is less than 30 ml/min/1.73 m^2^ and is not recommended when eGFR is between 30 and 45 ml/min/1.73 m^2^ (37, 180). Metformin should be stopped in cases of acute illness if there is a risk of dehydration, poor fluid intake, and hypoxemia, such as sepsis (179). Additionally, Metformin should be withheld before surgery and restarted once oral intake has resumed (37, 179). Metformin should also be discontinued before administration of radiographic contrast and for 48 hours afterwards until renal function is re-evaluated (37, 179).

## Metformin treatment to prevent T2D

Since T2D is a severe and aggressive metabolic disorder in children, prevention efforts have targeted the upstream events in its development, including pre-diabetes (181) (**Table 2**).

The current worldwide pooled prevalence of pre-diabetes is 8.84% (182). Up to 8% of children and adolescents with pre-diabetes progress to T2D, with increased body mass index (BMI)-based measures being essential predictors of progression (183–185). While adult studies have shown that Metformin can help prevent the progression to T2D (186–188), most pediatric studies have focused on markers of insulin sensitivity rather than T2D development as an outcome.

The effect of Metformin on markers of glycemic control or insulin resistance, including fasting plasma glucose, HbA1c, fasting insulin, and HOMA-IR, has been variable. Trials that treated youth with obesity and clinical or biochemical signs of insulin resistance with Metformin provided mixed results. Some studies demonstrated a reduction in fasting plasma glucose and insulin levels with an improvement in insulin sensitivity; yet, there were no differences in insulin levels or HOMA-IR in other studies (115, 116, 189–191). In a small, randomized controlled trial that included children and youth with impaired glucose tolerance regardless of weight, Metformin treatment for three months reduced HbA1c and HOMA-IR (192). However, Metformin may not have a clear beneficial effect on β-cell preservation during puberty in children and adolescents with obesity and normoglycemia. A randomized controlled study that followed 44 normoglycemic children and adolescents with obesity from early puberty to puberty completion demonstrated that, although Metformin improved anthropometric parameters such as BMI z-score, waist circumference and percent body fat, it did not have a beneficial effect on insulin sensitivity or β-cell function compared to placebo (193).

The Metformin in Obese Children and Adolescents (MOCA) trial included children and adolescents with obesity and impaired glucose tolerance or hyperinsulinemia that were randomized to Metformin versus placebo. There was minimal improvement in fasting glucose levels at three months, and that trend was not persistent at six months (119). Similarly, in another randomized controlled trial that included children and adolescents with obesity and insulin resistance based on HOMA-IR, there was no significant difference in HbA1c or HOMA-IR when comparing Metformin to placebo over 18 months of therapy (133). Moreover, an open label 18-month extension of this study found that there was a progressive increase in both BMI and HOMA-IR in participants who continued Metformin (134).

In summary, while Metformin helps reduce the progression to T2D in adults and may positively influence markers of glycemic control and insulin sensitivity in children and adolescents, current evidence on the role of Metformin in preventing progression from pre-diabetes to T2D in youth is lacking. Adequately powered trials with long-term follow-up focusing on T2D prevention as a primary outcome in youth are required.

## Conclusion

Metformin is considered first-line therapy in newly diagnosed pediatric patients with T2D, with or without insulin. It is generally considered a well-tolerated and safe medication. Even though Metformin has been approved for use in children for almost three decades, long-term prospective studies in this population are lacking. They are needed to better understand Metformin’s role in reducing T2D complications and preventing progression from pre-diabetes to diabetes. In addition to improving glycemic control and insulin sensitivity, Metformin may support BMI reduction which is important in improving insulin sensitivity.

## Author contributions

MCS is the guarantor. The paper topics, layout, writing, editing, and final approval was done by both authors. All authors contributed to the article and approved the submitted version.
